# Development of an Artificial Intelligence–Based Automated Recommendation System for Clinical Laboratory Tests: Retrospective Analysis of the National Health Insurance Database

**DOI:** 10.2196/24163

**Published:** 2020-11-18

**Authors:** Md Mohaimenul Islam, Hsuan-Chia Yang, Tahmina Nasrin Poly, Yu-Chuan Jack Li

**Affiliations:** 1 Graduate Institute of Biomedical Informatics, College of Medical Science and Technology Taipei Medical University Taipei Taiwan; 2 International Center for Health Information Technology Taipei Medical University Taipei Taiwan; 3 Research Center of Big Data and Meta-analysis, Wan Fang Hospital Taipei Medical University Taipei Taiwan; 4 Department of Dermatology Wan Fang Hospital Taipei Taiwan; 5 TMU Research Center of Cancer Translational Medicine Taipei Medical University Taipei Taiwan

**Keywords:** artificial intelligence, deep learning, clinical decision-support system, laboratory test, patient safety

## Abstract

**Background:**

Laboratory tests are considered an essential part of patient safety as patients’ screening, diagnosis, and follow-up are solely based on laboratory tests. Diagnosis of patients could be wrong, missed, or delayed if laboratory tests are performed erroneously. However, recognizing the value of correct laboratory test ordering remains underestimated by policymakers and clinicians. Nowadays, artificial intelligence methods such as machine learning and deep learning (DL) have been extensively used as powerful tools for pattern recognition in large data sets. Therefore, developing an automated laboratory test recommendation tool using available data from electronic health records (EHRs) could support current clinical practice.

**Objective:**

The objective of this study was to develop an artificial intelligence–based automated model that can provide laboratory tests recommendation based on simple variables available in EHRs.

**Methods:**

A retrospective analysis of the National Health Insurance database between January 1, 2013, and December 31, 2013, was performed. We reviewed the record of all patients who visited the cardiology department at least once and were prescribed laboratory tests. The data set was split into training and testing sets (80:20) to develop the DL model. In the internal validation, 25% of data were randomly selected from the training set to evaluate the performance of this model.

**Results:**

We used the area under the receiver operating characteristic curve, precision, recall, and hamming loss as comparative measures. A total of 129,938 prescriptions were used in our model. The DL-based automated recommendation system for laboratory tests achieved a significantly higher area under the receiver operating characteristic curve (AUROCmacro and AUROCmicro of 0.76 and 0.87, respectively). Using a low cutoff, the model identified appropriate laboratory tests with 99% sensitivity.

**Conclusions:**

The developed artificial intelligence model based on DL exhibited good discriminative capability for predicting laboratory tests using routinely collected EHR data. Utilization of DL approaches can facilitate optimal laboratory test selection for patients, which may in turn improve patient safety. However, future study is recommended to assess the cost-effectiveness for implementing this model in real-world clinical settings.

## Introduction

Laboratory tests are key components of the health care system and patient safety [1]. These tests assist physicians, helping them make many important decisions related to prevention, diagnosis, treatment, and management of chronic diseases [2]. However, in recent years, laboratory error rates have increased significantly, which has raised serious concerns about patient’s safety. Compared with other types of medical errors, laboratory errors have received little attention, despite these errors often causing significant harm to the patients [3]. Previous studies have reported that indiscriminate and inappropriate use of laboratory tests puts a significant and unnecessary burden on the health care system [4,5]. The value and associated cost of such inappropriate tests in the diagnostic and management process thus need to be determined.

Inappropriate testing can be in several forms. The first one is overutilization or overordering, which refers to recommended tests to the patients that are ordered without any indication. The second one is underutilization, which refers to recommended laboratory tests that are indicated but not ordered. Overutilization can result in unnecessary blood draws and other sample-collection procedures [6,7]. It increases the likelihood of false-positive results, which can lead to incorrect diagnoses, increased costs, and potential harm due to unwarranted additional intervention [8]. By contrast, underutilization can result in morbidity due to delayed or missed diagnoses and in downstream overutilization. Both overutilization and underutilization can lead to longer hospital stays and contribute to legal liability.

Deep learning (DL), a subset of machine learning, is being used in many areas including health care and has already shown its promise in various domains. This success can be attributed to an increase in computational power and the availability of massive amounts of data sets [9,10]. The field of DL has achieved immense success in training the machines to understand and manipulate data, including images [11], language [12], and speech [13]. In particular, health care and medicine are reaping significant benefits from the field because of the sheer volume of data generated every day in different forms. A quick and accurate laboratory test is crucial for patient’s safety through successful diagnosis and proper treatment of diseases. Because DL algorithms can easily handle hundreds of thousands of attributes and are capable of detecting and utilizing their interaction, developing an automated recommendation tool is always appreciable to improve proper clinical decisions. Accordingly, our study developed and evaluated a DL algorithm–based automated recommendation system using variables available in electronic health records (EHRs). We hypothesized that the DL algorithm can capture high-dimensional, nonlinear relationships among clinical features and a laboratory test recommendation system can be developed that can help physicians prescribe laboratory tests to individual patients more accurately as well as ensure safety of these patients.

## Methods

### Data Sources

We collected data from the Taiwanese National Health Insurance Research and Development (NHIRD) database, which contains all claims for the medications and diagnoses data of 23 million (covers approximately 99.9% of the total population in Taiwan) Taiwanese. The database includes patients’ demographic information, number of prescriptions, the brand and generic name of the drugs, the date of prescriptions, dosage of medication, and diagnosis. The quality and completeness of this database are excellent and have been used to conduct high-quality research [14-16]. This study was approved by the Taipei Medical University Research Ethical Board. Participants’ consent was not required because all the individual information was deidentified.

### Study Population

In this study, we retrieved prescription information for those patients who visited the cardiology department at least once from 2 million randomly selected patient’s data from the NHIRD database between January 1, 2013, and December 31, 2013.

### Variables Collection and Data Cleaning

We collected EHR data available at the time of ordering laboratory tests to develop the predictive model; these data included patients’ demographics, visit date, department ID, diagnosis, medications, and laboratory tests. We considered the first 3 digits in ICD-9-CM to retrieve information about comorbidities. The ICD-9-CM is usually distributed from 001 to 999 and V01 to V82. Furthermore, we considered the first 5 characters of the ATC code that cover almost every medication in a single category. For example, the 5 digits ATC of the code C09AA (ACE inhibitors, plain) include all plain ACE inhibitors such as C09AA01 (captopril), C09AA02 (enalapril). However, 7 characters (e.g., R06AX12) were considered for other drugs with “X” as the fifth character because usually “X” means other agents in the ATC code. The overall data set retrieved included 328 types of laboratory tests. This is a large amount of data and most laboratory tests were not ordered frequently, which can make prediction performance worse. We therefore calculated the percentage of all laboratory tests and selected a threshold of 0.5% to be included in this study. Finally, we narrowed the laboratory tests down to 35, which contributed to at least 0.5% of all tests in the study period. However, these 35 tests contributed to more than 90% of total tests (see Table S2 in Multimedia Appendix 1). All extracted data were used to make the matrices for data normalization (see Table S1 in Multimedia Appendix 1), and then used to train the deep neural network (DNN)–based multilabel prediction model to correctly identify laboratory items.

### Model Development and Validation

**Figure 1:**
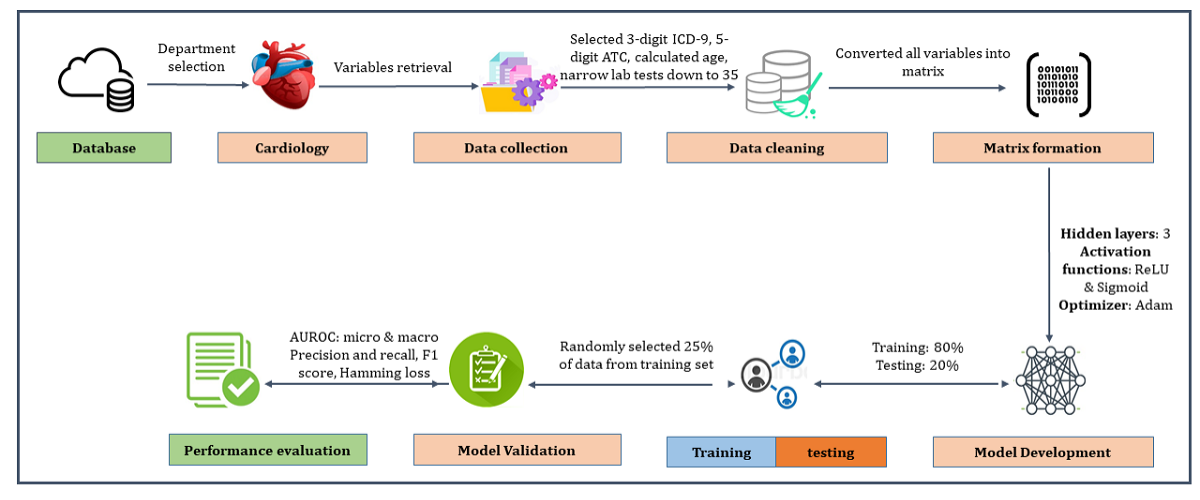
Overall study design.

**Figure 2:**
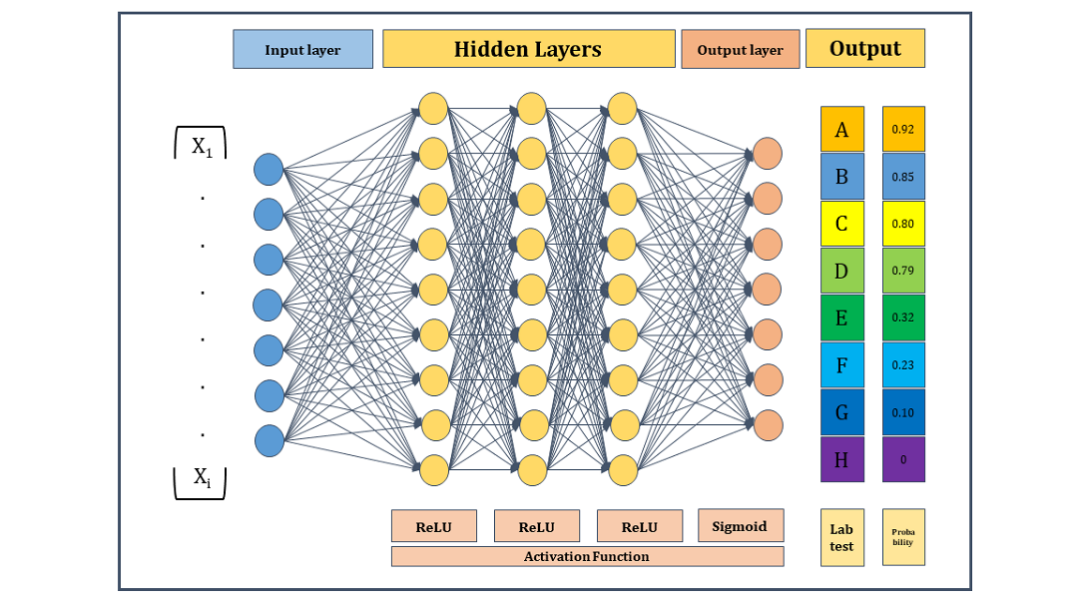
An architecture of proposed deep learning model.

In this study, 80% of data were assigned to the training set, and 20% to the testing set. In the internal validation, we randomly selected 25% of the data from the training set and evaluated model performance (Figure 1). We developed the DNN model on the training set using all variables and assessed the model using the validation set to predict laboratory tests (see Figures S1 and S2 in Multimedia Appendix 1). DNN is an algorithm in which an artificial neural network consists of multiple layers between the input layer and the output layer. The input of DNN moves through the layers calculating the probability of each output (Figure 2). We used 3 hidden layers. Activation functions used in this model were ReLU and Softmax. We used 20 epochs in our model.

#### Activation Function

The activation function is an integral part of a neural network and does the nonlinear transformation (ie, it describes the input and output relations in a nonlinear way). However, it is this nonlinearity element that allows for higher flexibility and performing complex tasks during the whole model learning process. It helps to speed up the whole learning process. Several activation functions such as sigmoid or ReLU are commonly used in practice.

##### Sigmoid Function

This function takes a real-value input and converts it into a range between 0 and 1. The sigmoid function is defined as follows:

σ(x) = 1/(1+e^–x^) **(1)**

Here it is clear that it will convent the output between 0 and 1 when the input varies in (–∞, ∞). A neuron can use the sigmoid for computing the nonlinear function σ(y = wx + b). If y=wx + b is very large and positive, then e^–y^ → 0, so σ(y) → 1, whereas if y = wx + b is very large and negative, then e^–y^→∞, so σ(y) → 0.

##### ReLU

It is called the rectified linear unit and takes a real input variable and thresholds it at zero (ie, replace native values with zero). The ReLU function is defined as follows:

f(x) = max (0, x) **(2)**

#### Optimization Algorithms

**Figure 3:**
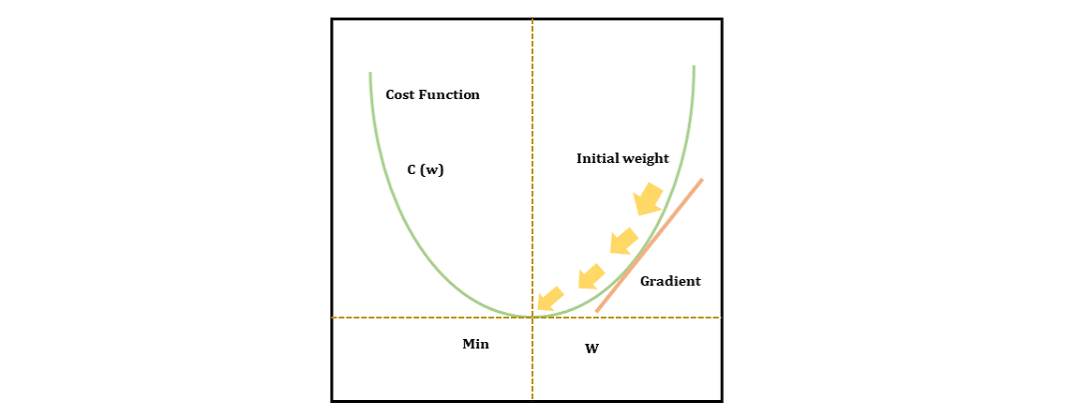
The process of gradient descent.

These algorithms are generally used to minimize errors and generate slightly better and faster results by updating input parameters such as weight and bias values. *Gradient descent* is the most widely used optimization algorithm that helps us understand whether the function is decreasing or increasing at a particular point (Figure 3).

The cost function C is the initial value and the desired point is C_min_. The starting weight is w_0_, with each step presented as r while the gradient represents the direction of maximum increase. The direction of the value can be expressed mathematically as the partial derivative ac/∂w to evaluate the time needed for w to reach step r, whereas the opposite direction can be expressed as –(ac/∂w)(w_r_)_._ The most commonly used optimizers are Momentum, Adagrad, AdaDelta, Adam.

### Performance Evaluation

We assessed the performance of the DNN model on the validations set for laboratory test recommendations using the following metrics.

#### Micro-AUC

This averages the prediction matrix. S_micro_ corresponds to a set of correct quadruples. The formula for calculating micro–area under the curve (micro-AUC) is

Micro-AUC = (|S_micro_|)/[(Σ^m^_i=1_|Y^+^_i._|)·(Σ^m^_i=1_|Y^–^_i._|)] **(3)**

Smicro = {(a,b,i,j)|(a,b)∈Y^+^_.i_ × Y^–^_.j_, f_i_(x_a_) ≥ f_j_(x_b_)} **(4)**

#### Macro-AUC

This averages each label. S_micro_ corresponds to a set of correctly ordered instance pairs on each label. The formula for calculating macro-AUC is

Macro-AUC = (1/l)Σ^l^_j=1_(|S^j^_macro_|)/(|Y^+^_.j_||Y^–^_.J_|) **(5)**

S^j^_macro_ = {(a,b)}∈Y^+^_.j_ × Y^–^_.j_|f_i_(x_a_) ≥ f_i_(x_b_)| **(6)**

#### Micro-F1

This averages the prediction matrix, and is calculated as follows:

Micro-F1 = (2Σ^l^_j=1_Σ^m^_i=1_y_ij_h_ij_)/(Σ^l^_j=1_Σ^l^_i=1_y_ij_ + Σ^l^_j=1_Σ^m^_i=1_h_ij_) **(7)**

#### Macro-F1

It averages each label, and is calculated as follows:

Macro-F1 = (1/l)Σ^l^_j=1_(2Σ^m^_i=1_y_ij_h_ij_)/(Σ^m^_i=1_y_ij_ + Σ^m^_i=1_h_ij_) **(8)**

#### Average Precision

This reflects the average fraction of relevant labels ranked higher than one other relevant label, and is calculated as follows:

Average precision = (1/m)Σ^m^_i=1_[1/(|y_i._^+^|)Σ_j∈Y_^+^_i._[|S^ij^_precision_|/rankF(x_i_,j)] **(9)**

S^ij^_precision_ = {k ∈ Y^+^_i._|rank_F_(x_i_,k) ≤ rank_F_(x_i_,j)|} **(10)**

#### Hamming Loss

It is the most commonly used metric to evaluate the performance of a multilabel classifier. It is the average symmetric difference between a set of true labels and a set of predicted labels of the data set. Its formula is as follows:

hloss (H) = (1/ml)Σ^m^_i=1_Σ^l^_j–1_[[h_ij_≠y_ij_]] **(11)**

The hamming loss (HL) value ranges from 0 to 1. A lesser value of HL indicates a better classifier.

## Results

### Prescriptions

In this study, we considered all patients who visited the cardiology department. A total of 37,890 patients visited the department at least once between January 1, 2013, and December 31, 2013. The number of male patients was higher than the number of female patients (51.11% [19,366/37,890] vs 48.89% [18,524/37,890]) and the age of patients ranged from 4 to 102 years. A total of 129,938 prescriptions with laboratory tests were ordered in the cardiology department (Table 1).

**Table 1:** Characteristics of patients and clinical variables.

Variables		Values	
Total number of prescription		129,938	
Total number of patients		37,890	
Age (years), range		4-102	
**Gender**			
	Male, n (%)		19,366 (51.11)
	Female, n (%)		18,524 (48.89)
Number of drugs input		416	
Number of diseases input		714	
Number of laboratory tests		35	

### Prediction of Laboratory Tests

**Figure 4:**
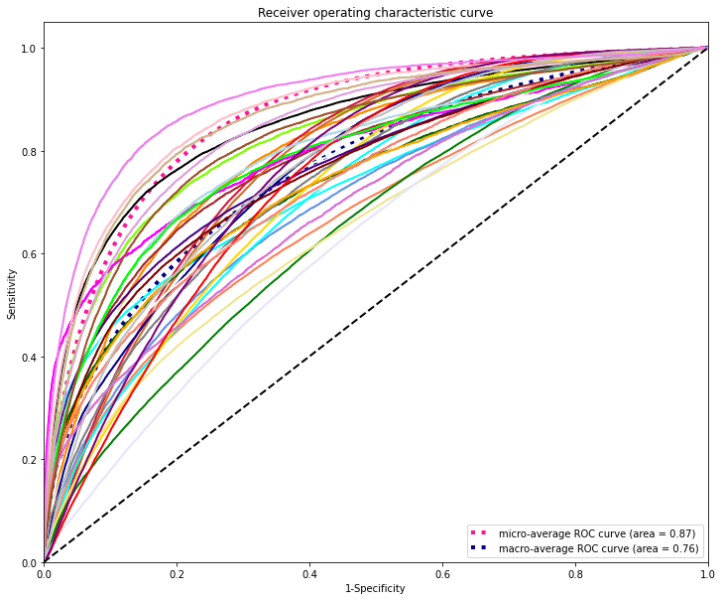
Receiver operating characteristic (ROC) curves of the deep learning model for predicting laboratory tests.

A total of 1132 input variables were used to predict the 35 types of laboratory tests. The DL model was applied to data from the cardiology department to predict laboratory tests accurately; the model achieved good discrimination (AUROC_macro_=0.76 and AUROC_micro_=0.87). Figure 4 shows the area under the receiver operating characteristic curve (AUROC) by the DL model. The range of the AUROC was 0.63-0.90 (see Figure S3 and Table S3 in Multimedia Appendix 1).

The DL model’s precision, recall, F1 score, and HL based on varying cutoffs for clinical laboratory test prediction are presented in Table 2. Precision, recall, F1 score, and HL ranged from 24% to 56%, 67% to 99%, 36% to 55%, and 0.16 to 0.46, respectively.

**Table 2:** Recall, precision, F1 score, and hamming loss of the model based on varying cutoffs for clinical laboratory test prediction.

Cutoffs	Recall^a^	Precision^a^	F1 score^a^	Hamming loss
0.01	0.99	0.24	0.36	0.46
0.05	0.94	0.33	0.45	0.39
0.10	0.89	0.40	0.50	0.29
0.15	0.85	0.44	0.52	0.24
0.20	0.80	0.47	0.54	0.21
0.25	0.76	0.51	0.55	0.19
0.30	0.71	0.54	0.55	0.17
0.35	0.67	0.56	0.55	0.16

^a^Overall (micro and macro) result presented.

## Discussion

### Principal Findings

In this study, we developed and validated a DL-based automated model to recommend laboratory tests based on individual patient clinical history. To our knowledge this is the first study which evaluated the performance of a DL algorithm to recommend laboratory tests, and achieved good performance; therefore, this model can be used in a real-world clinical setting. The main advantage of this model is that it requires minimal input data such as gender, age, disease, and drug information, and thus can be easily integrated into EHR systems. Most importantly, the model can be adjusted for different cutoff values according to physician needs. Moreover, physicians can select the required laboratory tests for an individual patient from a provided list of laboratory tests. This would ensure performing a quick and accurate test. The model showed high discrimination capacity; hence, implementation of this model would ensure accurate laboratory tests, improve patients’ safety, and reduce unnecessary costs associated with wrong orders.

### Comparison With Other Study

Previously, Wright et al [17] developed an association and data mining technique to suggest laboratory tests. Using a support threshold of 5 and a confidence threshold of 10%, there were 5361 associations between disease and laboratory tests. They reported a higher accuracy (55.6%) across the top 500 associations. The main problems of the system were that the relationships identified were indirect, one to one (drugs and laboratory or disease and laboratory), and had pseudo associations (metformin and hypertension). Furthermore, if the patients had multiple drugs, diseases, and laboratory tests, then their model was unable to find solutions and creates many associations. However, in a real clinical setting, one patient can be suggested to undergo multiple laboratory tests in 1 prescription. For example, a patient with diabetes could have been given multiple tests at the same time. Our model showed a higher accuracy (0.85) to predict laboratory tests. Moreover, our multilabel prediction model could provide a list of laboratory tests based on patients’ clinical history; therefore, physicians could choose laboratory tests from provided lists.

### Clinical Implications

Health care budgets worldwide are facing increasing pressure to minimize costs while maintaining quality care and ensuring patients’ safety [18]. The laboratory tests are often considered a central part of controlling health expenditures and ensuring patients’ safety. A previous study in the UK reported that pathology investigations are involved in 70%-80% of all health care decisions and cost the UK National Health Service (NHS) £2.5 billion (US $4 billion) annually [19]. Proper utilization of limited resources and curbing numerous unnecessary laboratory tests will help reduce health care costs because approximately 2.9%-56% of all laboratory tests are reported to be likely overutilized [20]. About 30% of the outpatient laboratory tests were found to be inappropriate and ordered just for patient check-ups [21]. However, inappropriate testing can contribute to increasing patient anxiety, iatrogenic anemia, and patient dissatisfaction.

Several groups of researchers have proposed many ways to control inappropriate laboratory test ordering, but it remains unclear which is the most effective or how to integrate these ways with other systems designed to control laboratory costs. Some have suggested reducing the reimbursement rate to control expenditures on laboratory services. Although this approach can be effective in the short run, it has several fundamental flaws. The second approach is linked to medical necessity; laboratory test cost can be reduced by decreasing the utilization of tests that are not medically necessary; however, it is very difficult to define the appropriate use of laboratory tests. Albeit significant progress has already been made, much work remains to be done in this area. A third approach has been active management of test utilization by laboratory staff. This approach has been used mostly in academic medical centers, often integrated as part of training for residents and fellows [22]. It can also vary from simply having laboratory staff act as gatekeepers for specific tests to more robust, systematic methods for improving test utilization [4]. However, our automated laboratory tests recommendation system is based on real-world clinical data and showed high discrimination capabilities. This model can thus help reduce unnecessary health care costs by recommending exact and appropriate laboratory tests based on individual patient problems.

### Strengths and Limitations

Our study has several strengths that need to be addressed. (1) This is the first study to evaluate and utilize the DL model to recommend laboratory tests using variables available in EHR. This study can therefore be used as a benchmark for future studies. (2) Our novel model is significantly more accurate and can adjust the cutoff value according to physician demand. Third, our evaluation of DL algorithms was rigorous, including fewer variables, and the model was developed based on daily clinical practice data.

Our study has also several limitations: (1) Our model was developed based on data from only the cardiology department; however, this model can be extended for use in other departments using their own data. (2) This model used only 35 laboratory tests in the prediction model; however, it covered more than 90% of total tests ordered. (3) Our model has not yet been tested using an external data set; however, we used internal validation to evaluate model performance. Sometimes performance of the model may deviate when it is validated using other data sets but this is not to a large extent.

### Future Perspective

**Figure 5:**
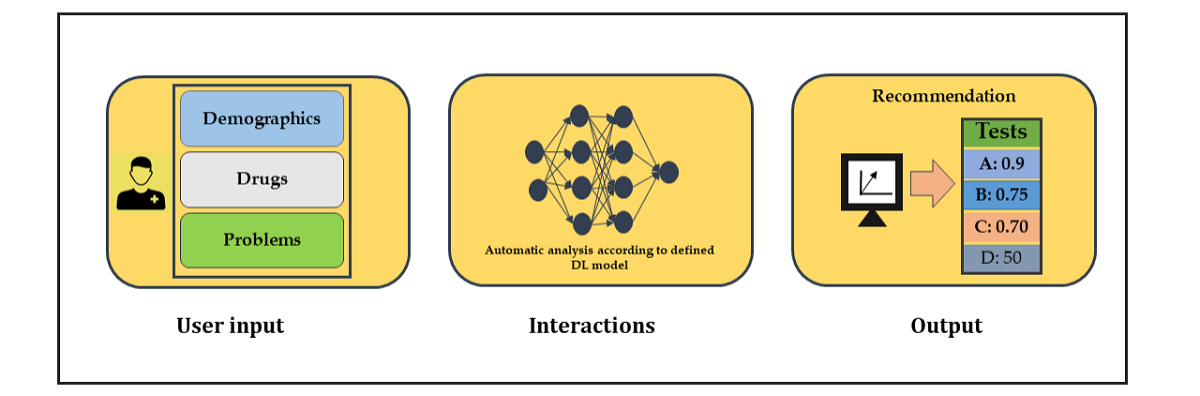
Proposed infographic of deep learning (DL)-based laboratory testing recommendation tool.

Our next step is to extend this work to other departments and includes using nonstructured data such as progress notes and operative notes. We believe that the inclusion of these data could increase our model performance. Moreover, we will use 10-year data to improve our model performance, although it would be computationally expensive. We also have a plan to include procedures in our system because it would further add value in the real-world clinical setting. Because our model showed higher sensitivity and a less false-positive rate, we will integrate our model with EHR to improve clinical decisions and reduce laboratory error rates. Although this would be quite powerful, it remains challenging for several reasons, including gold-standard evaluation and the acceptability of our model in clinical settings. However, one potential benefit of implementing this model in real-world clinical settings would be individual physician selection choice from a list of provided laboratory tests based on probability (Figure 5). It would not trigger many alerts and not hamper workflow.

### Conclusion

Using commonly available clinical variables, we developed and validated a DL algorithm that predicts laboratory tests with high accuracy, and recommends clinically relevant laboratory tests at the time of ordering. To our knowledge, this is the first study to evaluate the performance of algorithms and this predictive algorithm can serve as a clinical decision-support tool. Most importantly, our model could help reduce unnecessary laboratory test ordering and health care costs. The integration of this model into daily clinical practice may facilitate optimal laboratory test selection based on the appropriate thresholds. However, further research is necessary to assess the workflow of the system, and weigh the benefits of patients and physicians while implementing the model as an effective recommendation tool in clinical practice.

## Appendix

Multimedia Appendix 1 Artificial intelligence-based automated recommendation system for clinical laboratory tests.

## Abbreviations

ACE: angiotensin-converting enzyme
AUC: area under the curve
AUROC: area under the receiver operating characteristic curve
DL: deep learning
DNN: deep neural network
EHR: electronic health record
HL: hamming loss
NHIRD: National Health Insurance Research and Development
NHS: National Health Service
