# Supplementary material for: Development of an Artificial Intelligence–Based Automated Recommendation System for Clinical Laboratory Tests: Retrospective Analysis of the National Health Insurance Database
Source: JMIR Med Inform. 2020 Nov 18;8(11):e24163. doi: 10.2196/24163 (PMC7710445; doi:10.2196/24163)
Supplement: Multimedia Appendix 1 [file medinform_v8i11e24163_app1.docx]

**Supplementary**

**Artificial Intelligence-Based Automated Recommendation System for Clinical Laboratory Tests**

Md.Mohaimenul **Islam^1,2,3^**, Hsuan Chia **Yang^1,2,3^**, Tahmina Nasrin **Poly^1,2,3^**, Yu-Chuan **(Jack) Li^1,2,3,4,5*^**

**1. Model building:**

We developed an DNN model to predict laboratory test items for particular patients. Originally developed as mathematical theories of the information-processing activity of biological nerve cells, the structural elements used to describe an DNN are conceptually analogous to those used in prediction model/ recommendation model, despite it belonging to a class of statistical procedures. We used three hidden layers and activation functions used in this model were ReLU and Softmax. The basic element of the model is given below:

**Perceptron:**

It is a simple algorithm that takes an input vector x of m values $(x_{1}, x_{2,}\ldots..x_{n})$ which is often recognized as input features or simply features. It then multiplies them by some factors called “weight”, represented by $w_{1,}w_{2},\ldots. w_{n}$, gives an outputs either 1 (yes) or 0 (no).

Mathematically expression of this function is given below:

$$f\left( x \right)=\left\{ \begin{aligned} 1 \\ 0 \end{aligned} \right.{wx+b>0 \atop otherwise}$$

Here, $w$ is a vector of weights, $wx$ is the dot product of $\sum_{j=1}^{m} w_{j}x_{j}$ , and $b$ is a bias. A weight is a connection between neuron that carries a value. However, the higher the value, the larger the weight is. However, $wx+b$ always defines a boundary hyperplane that changes position according to the values assigned to $w$ and $b$. If $x$ lies above the straight line, then the answer is positive, otherwise it is negative. Moreover,

$$f\left( x \right)=\left\{ \begin{aligned} 1 \\ 0 \end{aligned} \right.{if w_{1}x_{1}+ w_{2}x_{2}+\ldots+w_{i}x_{i}+ w_{n}x_{n}>b \atop otherwise}$$

The perceptron often takes some $linear combination of input values or features$ compare it to a $threshold value b$ and $return 1 if the threshold exceeded and zero if not.$

+∫

$$X_{1}$$

$$X_{2}$$

$$X_{n}$$

$$wx+b$$

**Figure S1:** Perceptron

**Multilayer perceptron (MLP)**:

An MLP (or Artificial Neural Network) is a deep, artificial neural network takes more than one perceptron. An MLP with three-layers, first layer consider as a $input layer$, and last consider as a $output layer$ and middle layer consider as a $hidden layer.$ $Input layer$ receive input value such as $x_{1}, x_{2}, \ldots.x_{n}$ and take the output ${(y}_{n})$ from the $output layer$. However, $number of hidden layer$ can be added according to need. An example of a MLP is given below:


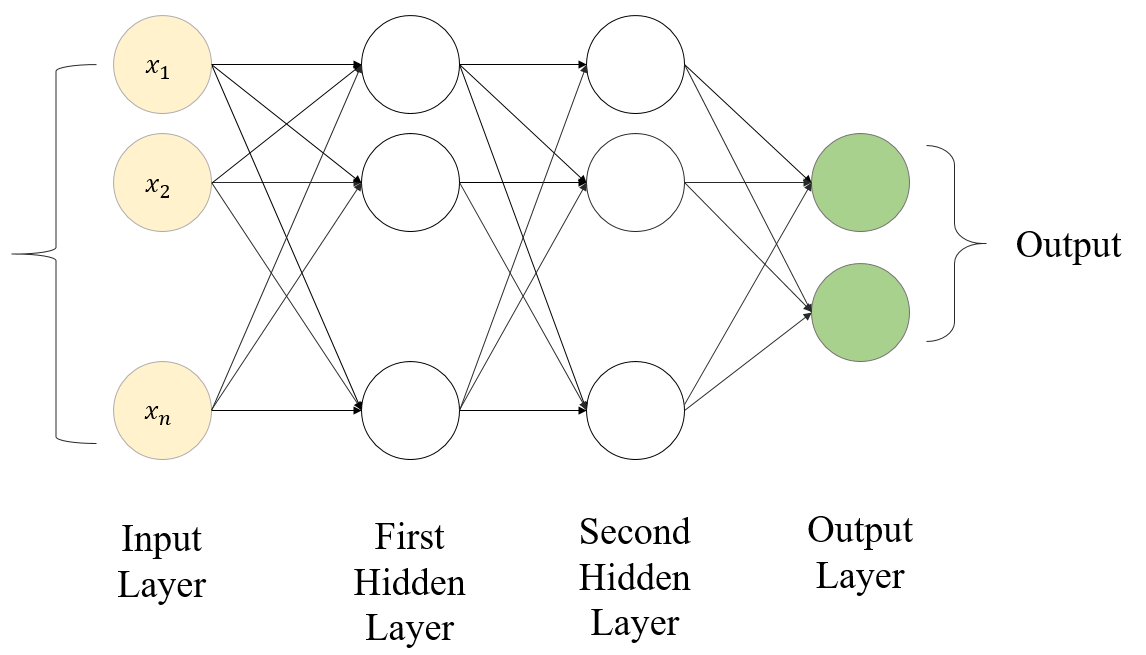


**Figure S2:** Basic structure of DNN model

It always applies to supervised learning problems in which a set of input and output variables are trained and learn the pattern between those in- and outputs. However, training process involves with adjusting the input parameters or the weights and biases. Because, all the edge weights are randomly assigned initially. The adjustment process need to be repeated until the output error is below or equal to a predetermined threshold. It is, however, important to minimize the errors during the training process. To minimize the errors during the training process, $backpropagation$is used to adjust those weight and biases to make less errors. The error can be measured in a variety of ways such as $root mean squared error (RMSE)$, $mean bias error \left( MBE \right)$.

In the classification outcome, activation function like $softmax function$ is used in the $output layer$ to make sure that the outputs are probabilities and they take value up to 1. An arbitrary real-value score is always taken by the $Softmax activation$function and it then convert it to a vector values between zero and one. It is like-

$$P\left( A \right)+P\left( B \right)=1$$

**Multi-label classification**: Multi-label classification and the strongly related problem of multi-output classification are variants of the classification problem where multiple labels may be assigned to each instance. Multi-label classification is a generalization of multiclass classification, which is the single-label problem of categorizing instances into precisely one of more than two classes; in the multi-label problem there is no constraint on how many of the classes the instance can be assigned to. Difference between multi-class classification & multi-label classification is that in multi-class problems the classes are mutually exclusive, whereas for multi-label problems each label represents a different classification task, but the tasks are somehow related. Classification task with more than two classes. Each sample can only be labelled as one class.

For example, classification using features extracted from a set of patients with laboratory tests, where each patient may either be of a glucose, urine, or Na. Each patient is one sample and is labelled as one of the 3 possible classes. Multiclass classification makes the assumption that each sample is assigned to one and only one label - one sample cannot, for example, be both a glucose and a urine.

**Table S1**: An example of patient data with multiple laboratory test

| **id** | **id_sex** | **id_dob** | **visit date** | **icd** | **drugs** | **Lab tests** |
| --- | --- | --- | --- | --- | --- | --- |
| **1** | **Female** | **19540912** | **2013.01.23** | **272** | **A02BA** | (09001C, 09004C, 09005C, 09043C) |
|  |  |  |  |  | **M01AE** | (09001C, 09004C, 09005C, 09043C) |
|  |  |  |  |  | **M03BB** | (09001C, 09004C, 09005C, 09043C) |
|  |  |  |  | **533** | **A02BA** | (09001C, 09004C, 09005C, 09043C) |
|  |  |  |  |  | **M01AE** | (09001C, 09004C, 09005C, 09043C) |
|  |  |  |  |  | **M03BB** | (09001C, 09004C, 09005C, 09043C) |
|  |  |  |  | **723** | **A02BA** | (09001C, 09004C, 09005C, 09043C) |
|  |  |  |  |  | **M01AE** | (09001C, 09004C, 09005C, 09043C) |
|  |  |  |  |  | **M03BB** | (09001C, 09004C, 09005C, 09043C) |

**Matrix formation**: Transform between inerrable of timetables and a multilabel format. Although a list of sets or tuples is a very intuitive format for multilabel data, it is unwieldy to process. This transformer converts between this intuitive format and the supported multilabel format: a (samples x classes) binary matrix indicating the presence of a class label.

Formula for converting multi-label data into matrix is given below:

*from sklearn. preprocessing import MultiLabelBinarizer*

*mlb = MultiLabelBinarizer ()*

*mlb.fit_transform ([{'09001C', '09004C'}, {'09005C '}])*

*array ([[0, 1, 1],*

*[1, 0, 0]])*

**Laboratory tests:**

**Table S2: List of laboratory tests**

| **Lab code** | **Test name** |
| --- | --- |
| 09005C | Glucose |
| 09015C | Creatinine (B) CRTN |
| 09026C | S-GPT/ALT |
| 09004C | Triglyceride |
| 09001C | Cholesterol, total |
| 09022C | K(Potassium) |
| 09044C | LDL-C (Lowdensity lipoprotein cholesterol) |
| 08011C | CBC-I(WBC,RBC,HB,HCT,PLATELET COUNT,MCV,MCH,MCHC) |
| 09006C | HbA1c (Hemoglobin A1c) |
| 09002C | BUN, blood urea nitrogen |
| 09013C | Uric acid |
| 09021C | Na (Sodium) |
| 09025C | S-GOT/AST |
| 09043C | HDL-C (highdensity lipoprotein cholesterol) |
| 08013C | WBC differential count |
| 06012C | General urine examination |
| 08026C | Prothrombin time |
| 09032C | CPK(Creatine-phospho-kinase) |
| 12015C | C.R.P (C-reactive protein) - Nephelometry |
| 09071C | CK-MB (Creatine phosphokinase-MB) |
| 08003C | Hemoglobin (Hb) |
| 09038C | Albumin |
| 09016C | Creatinine (U) CRTN |
| 12111C | Microalbumin (Nephelometry) |
| 09011C | Ca (Calcium) |
| 09029C | Bilirubin total |
| 08036B | APTT (activated partial thromboplastin time) |
| 09040C | Total protein |
| 09027C | Alkaline phosphatase |
| 09041B | Blood gas analysis |
| 09112C | TSH(EIA/LIA) |
| 08002C | W.B.C |
| 09012C | P (Phosphoras) |
| 13007C | Common anaerobic culture |
| 08004C | Hematocrite (Hct) |


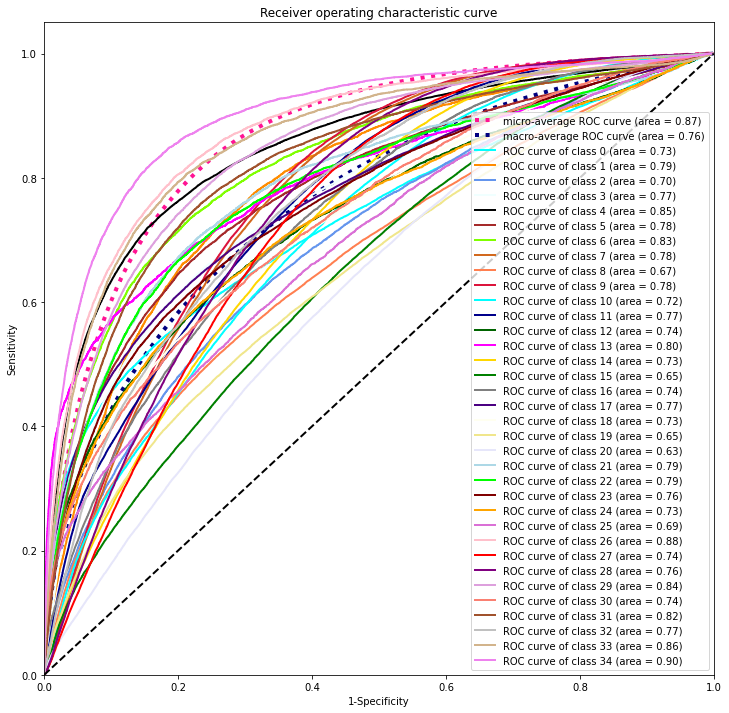


**Figure S3:** The ranged of the Operating characteristic area under the curve was 0.63-0.90

**Table S3**: AUROC for different laboratory tests.

| Label | Laboratory tests name | AUROC |
| --- | --- | --- |
| Class 0 | General urine examination | 0.73 |
| Class 1 | W.B.C | 0.79 |
| Class 2 | Hemoglobin (Hb) | 0.70 |
| Class 3 | CBC-I(WBC,RBC,HB,HCT,PLATELET COUNT,MCV,MCH,MCHC) | 0.77 |
| Class 4 | WBC differential count | 0.85 |
| Class 5 | Prothrombin time | 0.78 |
| Class 6 | APTT (activated partial thromboplastin time) | 0.83 |
| Class 7 | Cholesterol, total | 0.78 |
| Class 8 | BUN, blood urea nitrogen | 0.67 |
| Class 9 | Triglyceride | 0.78 |
| Class 10 | Glucose | 0.72 |
| Class 11 | HbA1c (Hemoglobin A1c) | 0.77 |
| Class 12 | Ca (Calcium) | 0.74 |
| Class 13 | P (Phosphorus) | 0.80 |
| Class 14 | Uric acid | 0.73 |
| Class 15 | Creatinine (B) CRTN | 0.65 |
| Class 16 | Creatinine (U) CRTN | 0.74 |
| Class 17 | Na (Sodium) | 0.77 |
| Class 18 | K(Potassium) | 0.73 |
| Class 19 | S-GOT/AST | 0.65 |
| Class 20 | S-GPT/ALT | 0.63 |
| Class 21 | Alkaline phosphatase | 0.79 |
| Class 22 | Bilirubin total | 0.79 |
| Class 23 | CPK(Creatine-phospho-kinase) | 0.76 |
| Class 24 | Albumin | 0.73 |
| Class 25 | Total protein | 0.69 |
| Class 26 | Blood gas analysis | 0.88 |
| Class 27 | HDL-C (high-density lipoprotein cholesterol) | 0.74 |
| Class 28 | LDL-C (Low-density lipoprotein cholesterol) | 0.76 |
| Class 29 | CK-MB (Creatine phosphokinase-MB) | 0.84 |
| Class 30 | TSH(EIA/LIA) | 0.74 |
| Class 31 | C.R.P (C-reactive protein) - Nephelometry | 0.82 |
| Class 32 | Micro albumin (Nephelometry) | 0.77 |
| Class 33 | Common anaerobic culture | 0.86 |
| Class 34 | Blood culture | 0.90 |
